# Alloying and Properties of C14–NbCr_2_ and A15–Nb_3_X (X = Al, Ge, Si, Sn) in Nb–Silicide-Based Alloys

**DOI:** 10.3390/ma11030395

**Published:** 2018-03-07

**Authors:** Panos Tsakiropoulos

**Affiliations:** Department of Materials Science and Engineering, University of Sheffield, Sheffield S1 3JD, UK; p.tsakiropoulos@sheffield.ac.uk

**Keywords:** intermetallics, Laves phase, A15 phase, alloying, hardness, creep

## Abstract

The oxidation of Nb–silicide-based alloys is improved with Al, Cr, Ge or Sn addition(s). Depending on addition(s) and its(their) concentration(s), alloyed C14-AB_2_ Laves and A15-A_3_X phases can be stable in the microstructures of the alloys. In both phases, A is the transition metal(s), and B and X respectively can be Cr, Al, Ge, Si or Sn, and Al, Ge, Si or Sn. The alloying, creep and hardness of these phases were studied using the composition weighted differences in electronegativity (∆χ), average valence electron concentrations (VEC) and atomic sizes. For the Laves phase (i) the VEC and ∆χ were in the ranges 4.976 < VEC < 5.358 and −0.503 < ∆χ < −0.107; (ii) the concentration of B (=Al + Cr + Ge + Si + Sn) varied from 50.9 to 64.5 at %; and (iii) the Cr concentration was in the range of 35.8 < Cr < 51.6 at %. Maps of ∆χ versus Cr, ∆χ versus VEC, and VEC versus atomic size separated the alloying behaviours of the elements. Compared with unalloyed NbCr_2_, the VEC decreased and ∆χ increased in Nb(Cr,Si)_2_, and the changes in both parameters increased when Nb was substituted by Ti, and Cr by Si and Al, or Si and Ge, or Si and Sn. For the A15 phase (i) the VEC and ∆χ were in the ranges 4.38 < VEC < 4.89 and 0.857 < ∆χ < 1.04, with no VEC values between 4.63 and 4.72 and (ii) the concentration of X (=Al + Ge + Si + Sn) varied from 16.3 to 22.7 at %. The VEC versus ∆χ map separated the alloying behaviours of elements. The hardness of A15-Nb_3_X was correlated with the parameters ∆χ and VEC. The hardness increased with increases in ∆χ and VEC. Compared with Nb_3_Sn, the ∆χ and hardness of Nb_3_(Si,Sn) increased. The substitution of Nb by Cr had the same effect on ∆χ and hardness as Hf or Ti. The ∆χ and hardness increased with Ti concentration. The addition of Al in Nb_3_(Si,Sn,Al) decreased the ∆χ and increased the hardness. When Ti and Hf, or Ti, Hf and Cr, were simultaneously present with Al, the ∆χ was decreased and the hardness was unchanged. The better creep of Nb(Cr,Si)_2_ compared with the unalloyed Laves phase was related to the decrease in the VEC and ∆χ parameters.

## 1. Introduction

The inherent temperature capability of Ni-based superalloys is limited by the melting point of Ni. Currently, new high temperature alloys are developed based on refractory metals. Nb–silicide-based alloys, also known as Nb in situ silicide composites, have the potential to meet property goals and satisfy environmental and performance targets for future aero-engines [1,2]. These alloys can have as many as 12 alloying additions, some of which are essential for improving their oxidation resistance and others for improving their strength and creep. These elements include Al (≤5 at %), Cr (≤8 at %), Fe (≤5 at %), Ge (≤5 at %), Hf (≤5 at %), Si (16–22 at %), Sn (≤5 at %) or Ti (10–26 at %) and refractory metals (Mo (≤8 at %), Ta (≤6 at %), W (≤4 at %)). Substitution of Si by Ge and Sn, and Cr by Fe has been studied [3,4,5]. Bewlay et al. reported that “additions of Fe were almost as effective as additions of Cr up to a concentration of 5 at %, and that the combination of Cr and Fe was no more effective than Cr alone for the same total concentration” [6]. In the microstructures of these materials, the bcc Nb solid solution(s) and Nb_5_Si_3_ silicide(s) can be stable together with the C14-NbCr_2_ Laves and A15-Nb_3_X (X = Al, Ge, Si, Sn) phases [3,4,7,8]. The latter two phases are considered to improve oxidation resistance [1,6,9]. The Laves phase usually forms in the last to solidify Cr rich melt in-between Nb solid solution (Nb_ss_) dendrites [7,8]. Depending on other alloying additions, the Laves phase can also surround the Nb_ss_ and protect it from oxidation. The A15 intermetallic phases form next to the Nb_ss_ but can form during oxidation below the scale at the substrate/scale interface [9,10]. Whether the A15 Nb_3_X phase(s) can form in the cast microstructure with/without Nb_ss_ and be stable with/without Nb_ss_ after exposure to high temperatures depends on the concentration(s) of element(s) X. The mechanical properties of the A15 phases are critical for the strength and creep of Nb–silicide-based alloys and for adhesion of their scale.

First, let us briefly discuss what is known about the alloying and properties of the NbCr_2_ Laves and A15-Nb_3_X intermetallic phases. The NbCr_2_ Laves phase belongs to the class of Frank–Kasper phases of general composition, AB_2_, with topological close packed (TCP) structures, where A is the largest atom and the closest packing of hard spheres is for the radius ratio r_A_/r_B_ = 1.225, where r_i_ is the radius of the metal atom, i (=A, B), in the stoichiometric composition of the phase [11]. The coordination numbers are 6 for the A atoms and 12 for the B atoms. The structure and stability of binary (unalloyed) and ternary Laves phases were reviewed by Stein et al. [12,13]. Binary Laves phases have homogeneity ranges extending to the A-rich or B-rich (or both) sides of the stoichiometric composition. Solubility is related to changes in the interatomic distance. For C14 Laves phases, a wider homogeneity range is observed for 1.12 < r_A_/r_B_ < 1.26 [11]. Extended solubility ranges have been attributed to anti-site atoms (for example, B atoms on A sites), vacancies and combinations of both. Anti-site defects form on both sides of the stoichiometric composition of NbCr_2_ [14].

Substitutional alloying elements can be added on one or both sites. Such substitutions can modify properties, for example, oxidation resistance. Despite the absence of binary Laves phases, ternary Laves phases can form with alloying—examples include Nb(Ni,Al)_2_ and Mo(Co,Si)_2_. There are no binary Laves phases based on Si. When a third element is added to a Laves phase, the solubility and site occupancies can vary significantly depending on the element. In the Nb–Cr binary the low and high temperature Laves phases, respectively, have cubic C15 and the hexagonal C14 structures [15]. The alloying possibilities of NbCr_2_ have been discussed by Shah and Anton [16]. The ternary C14 Nb(Cr,Si)_2_ is stabilised for Si ≥ 2 at %, when Si substitutes Cr in the NbCr_2_ [11,17]. The same is the case in which Cr in NbCr_2_ is substituted by Al [18,19]. Bardos et al. [17] attributed the Si effect in Laves phases (i.e., the stabilisation of the C14 Laves phase by Si) to the lowering of the electron concentration. Precipitation of cubic C15 (metastable phase) and hexagonal C14 (stable phase) (Nb,Mo)(Cr,Si,Al)_2_ Laves phases in the bcc phase of a Nb–27Mo–27Cr–9Al–9Si (at %) alloy during heat treatment has been reported [20]. The precipitation of a (Nb,Mo)(Cr,Fe,Si)_2_ Laves phase in a ferritic stainless steel contributed to its improved oxidation resistance [21].

The brittle to ductile transition temperature (DBTT) of the AB_2_ Laves phases is of the order of 0.65T_m_, where T_m_ is the melting temperature. Their hardness increases in the A sequence, Nb, Ta, for fixed B and in the B sequence, Cr, Fe, for fixed A, both of which correspond to increases in atomic number [22]. The brittleness of Laves phases has been linked with their structures and the high Peierls resistance to dislocation motion. The good oxidation resistance of NbCr_2_, which has been linked with low diffusion rates that have been attributed to its structure, has allowed its mechanical behaviour to be evaluated at temperatures up to 1673 K [22]. Creep resistance has been reported for NbCr_2_ [23]. Reported values for the hardness, toughness and yield stress of NbCr_2_ differ, owing to differences in the microstructures of the tested materials that have been attributed to the difficulty of making monolithic Laves phase ingots [22]. Experimental work has confirmed slip and twinning in C14 Laves phases [24]. The NbCr_2_, like other Laves phases, is brittle at low homologous temperatures. Monolithic NbCr_2_ has a low shear modulus G (≈ 80 GPa) and high Poisson’s ratio ν (0.34). High ν is an indication of a lack of strong directional bonding and a low G value suggests a potential for resistance to brittle failure (according to the Rice–Thomson criterion, if Gb/γ < 10 where γ is surface energy, there is a tendency for resistance to brittle failure). For NbCr_2_, the Gb/γ is about 6 for b = 1/6 <112>).

A significant number of intermetallics with melting temperatures above 1873 K have the A15 structure. Niobium can form the intermetallic phases Nb_3_Sn, Nb_3_Ge, Nb_3_Al, Nb_3_Si, Nb_3_Ga and Nb_3_Sb, all of which have the A15 structure with Cr_3_Si as a prototype. There is no data for Nb–silicide-based alloys with Ga and Sb alloying additions. The A15–Nb_3_Si is a metastable phase in the Nb–Si binary [25], and the other A15 phases are stable phases in their respective binary systems [15].

Many A15 intermetallics, for example Nb_3_Al, Nb_3_Ge, Nb_3_Sn, are adjacent to refractory metal solid solutions and are not line compounds. Furthermore, some of the transition metal alloying additions in Nb–silicide-based alloys form stable A15 phases, for example, Mo forms Mo_3_Al, Mo_3_Ge, Mo_3_Si and Mo_3_Sn, and Cr forms Cr_3_Ge and Cr_3_Si [15]. The A15 structure should have no octahedral interstitial sites and thus low solubility for interstitial elements, which is important for high temperature structural materials.

In A15–Nb_3_X, the X atoms form a bcc lattice and each cube face is bisected by orthogonal Nb chains. For example, in stoichiometric Nb_3_Sn, the distance between the Nb atoms is about 0.265 nm [26], i.e., slightly reduced compared with the shortest spacing between Nb atoms (about 0.286 nm), and this difference is linked with a very high DOS (density of states) near the Fermi level.

There are six Nb atoms and two Al atoms in the Nb_3_Al unit cell. All the Nb atoms are equivalent and all the Al atoms are equivalent. Alloying additions that substitute Nb strengthen the Nb_3_Al at high temperatures in the sequence W, Mo, Ta, Ti, and the stronger effect of the refractory metals has been attributed to their low diffusivities in it [27]. Alloying Nb_3_Al with Ti decreases covalent bonding [28]. The melting temperature of Nb_3_Al alloyed with Si is above 2473 K. Ti and Ta substitute Nb in the A15 lattice, and, as their concentration is increased, the A15 structure is stabilised [26,29].

The properties of unalloyed A15–Nb_3_X (X = Al, Ge, Si, Sn) intermetallics that can form in Nb–silicide-based alloys were studied in [28,30,31,32,33]. The moduli of elasticity of A15 intermetallics are not unusually high. Monolithic Nb_3_Al is brittle, has poor fracture toughness at ambient temperatures [34], and its yield strength is about 900 MPa at 1473 K [27].

The cubic symmetry of the A15 structure with its inherent isotropic coefficient of thermal expansion (CTE) is advantageous for cyclic oxidation. Plasticity has been observed in all A15 compounds above 1673 K. Compared with the peak hardness of Ni_3_Al at 873 K, Nb_3_Al has the same hardness at 1473 K, a temperature improvement of 600 degrees. The yield strengths of Nb_3_Al and Nb_3_Sn are comparable [35]. Crack free Cr_3_Si ingots can be produced with Sn additions, but Nb_3_Sn ingots are very brittle.

What is known about the NbCr_2_ Laves and A15 intermetallic phases that form in Nb–silicide-based alloys? Some of the alloying additions substitute Nb and others substitute Cr in NbCr_2_, or Nb and X are substituted in Nb_3_X; in other words, in Nb–silicide-based alloys, the C14 NbCr_2_-based Laves phases and A15 Nb_3_X-based phases are highly alloyed. The following compositions (at %) 26.4Nb–12Ti–5.8Mo–2.5Hf–0.9W–35.8Cr–8.2Si–5.5Al–1.6Sn–1.3Ge and 46.3Nb–11.3Ti–5.3Cr–7.8Ta–7.3W–0.3Hf–10.4Sn–5.2Si–4.8Al–1.3Ge are examples, respectively, of C14 NbCr_2_ based Laves and A15 Nb_3_X based phases in developmental Nb–silicide-based alloys.

Data concerning the alloying behaviours and properties of highly alloyed Laves and A15 phases is essential for the design of new Nb–silicide-based alloys. Data for the alloying behaviours of phases is provided in phase diagrams. Experimental data concerning the thermodynamic properties of Nb-based systems is limited [36]. Phase equilibria data is available for a small number of systems from thermodynamic modelling (CALPHAD) and ab initio calculations. There are disagreements between ternary phase diagrams of the same system. For example, for the Nb–Cr–Si system, there is disagreement about the phase equilibria between Nb_ss_, Nb_5_Si_3_ and NbCr_2_ Laves and about the liquidus projection [18,37,38,39]. There are also conflicting reports for the Nb–Ge binary [33]. The refractory metals, Mo, Ta and W, provide solid solution strengthening and improve the high temperature strength and creep of Nb–silicide-based alloys. Binary phase diagrams of refractory metals exist only for high temperatures (T > 2273 K) [15]. Phase equilibria data for Nb–Si based systems where C14 NbCr_2_ Laves and A15-Nb_3_X phases can form is insufficient. There are no phase diagrams for the Nb–Sn–Y (Y = Al, Cr, Fe, Hf, Mo, Ta, Ti, W) and Nb–Ge–Z (Z = Cr, Fe, Mo, Ta, W) systems.

Research to clarify discrepancies between existing phase diagrams [33,40] and to provide some of the missing data for ternary systems is underway. In the meantime, what can we learn about the alloying behaviours of the C14–NbCr_2_ Laves and A15–Nb_3_X phases in Nb–silicide-based alloys from data concerning the chemical compositions of these phases in developmental alloys? Can electronegativity (∆χ), valence electron concentration (VEC) and atomic size elucidate their alloying and differences between alloying additions as they did for tetragonal Nb_5_Si_3_ [41] and the bcc Nb solid solution(s) [42] in Nb–silicide-based alloys? Are changes in hardness and creep of these phases linked with changes in ∆χ and VEC as was the case for tetragonal Nb_5_Si_3_ [41]? The motivation for the work presented in this paper was to find answers to these questions.

The structure of the paper is as follows: First, the alloying of the C14–NbCr_2_ Laves phase is discussed using maps that are based on electronegativity, valence electron concentration and atomic size. Then, the same parameters are used to study of the alloying and hardness of A15 intermetallic phases. Finally, the creep of C14–NbCr_2_ Laves and A15 Nb_3_Al phases is compared.

## 2. Methodology, Results and Discussion

Experimental data concerning the actual average compositions of the C14–NbCr_2_ Laves and A15-Nb_3_X intermetallic phases in developmental Nb–silicide-based alloys [3,4,7,8,9,43,44,45,46,47,48,49,50,51,52,53] was used to find out whether there are relationships between the parameters ∆χ, VEC and atomic size. Phase stability can be considered in terms of *e*/*a* and VEC (number of valence electrons per atom filled into the valence band). The *e*/*a* ratio is a key parameter in the Hume–Rothery rules, and VEC is key to determining the Fermi level in the valence band. According to Mizutani [54], the *e*/*a* is difficult to use as a universal parameter in alloy design, because its value cannot be uniquely assigned to a transition metal, as it depends on the surrounding environment. Instead, VEC is a more important parameter in transition metal alloys.

The C14–NbCr_2_ Laves and A15–Nb_3_X intermetallic phases studied in this paper were in cast (AC) and/or heat treated (HT) microstructures of Nb–silicide-based alloys that were prepared in earlier research, meaning no new experimental data were created during the course of this study. As described elsewhere [7], the alloys were made using high purity (better than 99.99 wt %) elements and non-consumable (W) electrode arc melting in water cooled copper hearths and were heat treated in an Argon atmosphere at 1773 K for 100 h. The chemical composition of each alloy was determined in the AC and HT conditions using EPMA (electron probe microanalysis). The Vickers hardness measurements of A15 intermetallic phases in Nb–silicide-based alloys were done using a Mitutoyo micro-hardness testing machine. The load used was 0.1 kg and was applied for 20 s. At least 10 measurements were taken for each phase. The hardness measurements were taken from A15 phases in bulk microstructures, free of contamination by interstitials and with similar grain sizes. The creep data for the Laves phases and Nb_3_Al was taken from [23,55].

The actual average chemical composition (at %) of each phase, determined by EPMA, was used to calculate the aforementioned parameters using data for the elements from the same sources as in [42] and the equations given below. The parameter VEC was calculated using [VEC]_intermetallic_ = ∑_i_^n^*C_i_*(VEC)*_i_*, where C_i_ and (VEC)*_i_*, respectively, are the concentration (at %) and VEC of element i in the intermetallic. For the NbCr_2_ Laves phase, the electronegativity parameter was [∆χ]_Laves_ = ∑_i_^m^C_i_(χ_<Nb>i_) − ∑_i_^z^κ_i_(χ_<Cr>i_), where C_i_ and χ_<Nb>i_, respectively, are the concentration (at %) and Pauling electronegativity of Nb and element i substituting Nb in the Laves phase, and κ_i_ and χ_<Cr>i_, respectively, are the concentration (at %) and Pauling electronegativity of Cr and element i substituting Cr in the Laves phase. For the A15–Nb_3_X, the electronegativity parameter was [∆χ]_A15-Nb3X_ = ∑_i_^m^C_i_(χ_<Nb>i_*)* − ∑_i_^z^κ_i_(χ_<X>i_), where *C_i_* and χ_<Nb>i_, respectively, are the concentration (at %) and Pauling electronegativity of Nb and element i substituting Nb in the A15 phase, and κ_i_ and χ_<X>i_, respectively, are the concentration (at %) and Pauling electronegativity of X (=Al, Ge, Si, Sn) and element i substituting X in the A15 phase. For the Laves phase, R_<Nb>_ = ∑_i_^n^C_i_(r_<Nb>_)_i_, where *C_i_* and (r_<Nb>_)_i_, respectively, are the concentration (at %) and atomic radius of Nb and element i substituting Nb in the Laves phase, and R_<Cr>_ = ∑_i_^n^C_i_(r_<Cr>_)*_i_* is the concentration (at %) and atomic radius of Cr and element i substituting Cr in the Laves phase. For the A15 phase, <R> = ∑_i_^n^C_i_(r)_i_, where *C_i_* and (r)_i_, respectively, are the concentration (at %) and atomic radius of element i in the A15 phase.

### 2.1. C14–NbCr*_2_* Laves

In Nb–silicide-based alloys, the Laves phase that is stable in their microstructures is the hexagonal C14–NbCr_2_ [7,8,9,43,44,45,46,47,48,49,50,51], where Nb can be substituted by transition metals, such as Hf, Mo, Ta, Ti or W and Cr, by simple metal and metalloid elements (Al, Ge, Si, Sn). The stability of C14–NbCr_2_ in the microstructures of Nb–silicide-based alloys is in agreement with the literature about the effects of Al and Si on the structure of NbCr_2_. The substitution of Nb by the aforementioned transition metals is also in agreement with the literature [13].

The data for developmental Nb–silicide-based alloys showed (a) that the solubilities of the elements that substitute Nb in C14–NbCr_2_ were Hf ≤ 10.2 at %, 1.3 < Mo < 5.8 at %, 4.2 < Ta < 9.6 at %, 3.4 < Ti < 28.8 at % and W ≤ 5.2 at % (there is no chemical composition data for C14–NbCr_2_-based Laves phases where Cr is substituted by Fe in Nb–silicide-based alloys); (b) that in the alloyed NbCr_2_, the Cr concentration was in the range 35.8 < Cr < 51.6 at % and increased after heat treatment, from 35.8 < Cr < 49.2 at % to 44.7 < Cr < 51.6 at % and (c) that the solubilities of the elements that substituted Cr in the Laves phase were Al ≤ 11 at %, Ge ≤ 3.1 at %, 5 < Si < 12.6 at % and Sn ≤ 2.8 at %. Furthermore, if B = Al + Cr + Ge + Si + Sn, then the concentration of B in the cast and heat-treated microstructures, respectively, was in the ranges 50.9 < B < 62.4 at % and 53.1 < B < 64.5 at %, compared with 61 < Cr < 70 at % for unalloyed NbCr_2_ [15], which would suggest that homogeneity ranges on both the Nb and Cr sides of C14–NbCr_2_ formed in Nb–silicide-based alloys. The data for the concentration of B (=Al + Cr + Ge + Si + Sn) and the solubility of Si in C14 NbCr_2_ reported in [56] are in agreement with the aforementioned values.

**Figure 1:**
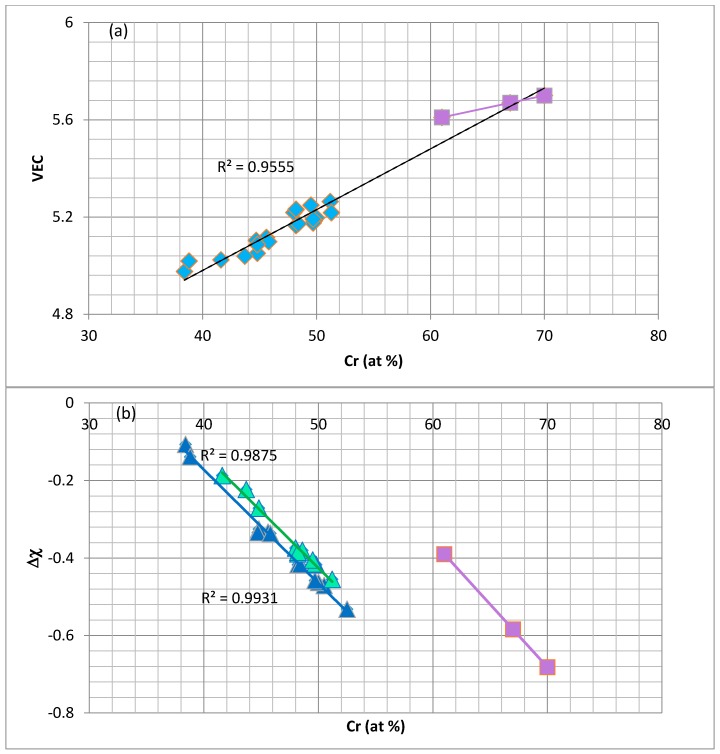
Valence electron concentrations (VEC) (**a**) and ∆χ (**b**) versus the Cr concentration in the C14 NbCr_2_ Laves phases. The squares represent the unalloyed NbCr_2_. In (**a**) the blue diamonds correspond to the same data as in (**b**). In (**b**), the data can be separated: the blue triangles represent Laves phases where Nb is substituted by Ti, Mo or Hf, and Cr is substituted by Si, Sn or Al, and the green triangles represent Nb, substituted by Ti and Hf, and Cr by Si, Al, Ge or Sn.

Unlike the tetragonal Nb_5_Si_3_ [41], no strong relationships were found between the concentrations of alloying elements in the C14–NbCr_2_ Laves phase. The alloying of C14–NbCr_2_ was studied using the parameters VEC, ∆χ, <R> = R_<Nb>_ + R_<Cr>_ and R_<Nb>_/R_<Cr>_. It should be noted that the latter ratio is not the same as the ratio r_A_/r_B_ (see introduction). Figure 1 shows how the parameters VEC and ∆χ change with the Cr concentration in NbCr_2_. Figure 1a shows data for alloyed Laves phases where Nb is substituted by Hf, Mo or Ti and Cr by Al, Ge, Si or Sn. The parameter VEC increases with the Cr concentration in the Laves phase, and the linear fit of data is very good (R^2^ = 0.9555). The extrapolated best fit line crosses the best fit line for the data for unalloyed NbCr_2_ and passes between the two data points for unalloyed NbCr_2_ with a high Cr content. Unlike the parameter VEC, the parameter ∆χ can separate the data for the Laves phase into two sets (see Figure 1b). In the latter, the data for unalloyed NbCr_2_ and two alloyed Laves phases falls on parallel lines and shows (i) that alloying makes ∆χ less negative and (ii) that as the Cr concentration in the Laves phase increases, the ∆χ becomes more negative.

**Figure 2:**
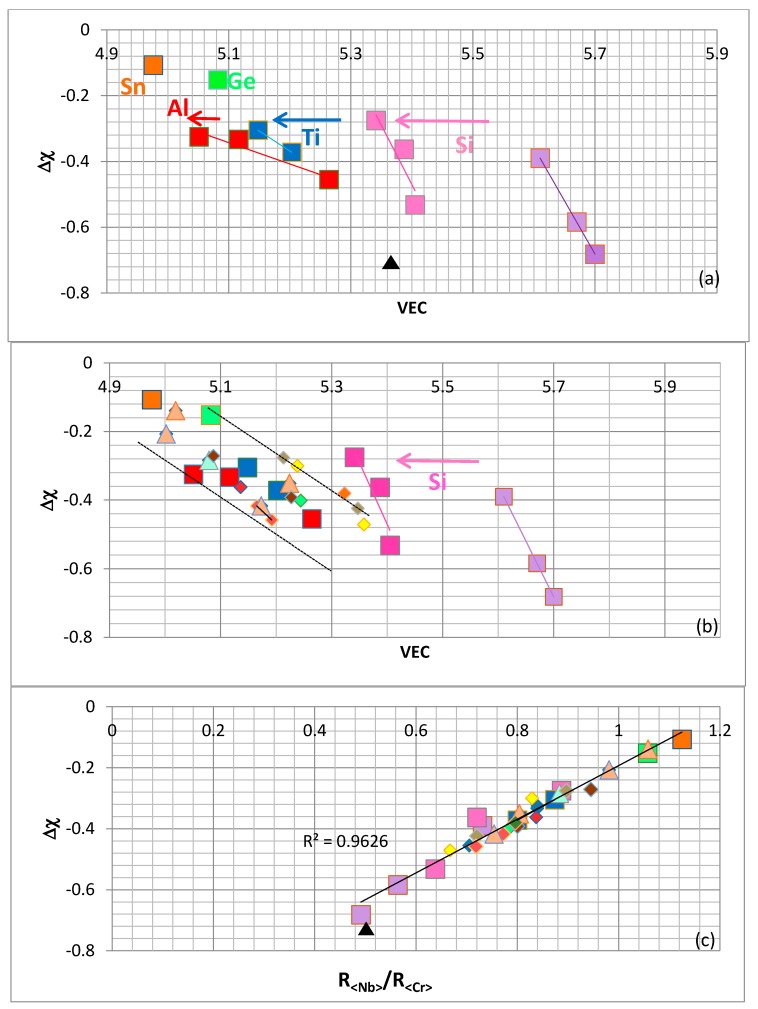
Laves phase ∆χ versus VEC maps are shown in (**a**,**b**), and (**c**) is the Laves phase ∆χ versus R<Nb>/R<Cr> map. In (**a**,**b**,**c**), the squares are data for NbCr_2_ (lavender), Nb(Cr,Si)_2_ (pink), (Nb,Ti)(Cr,Si)_2_ (blue), (Nb,Ti)(Cr,Si,Al)_2_ (red), (Nb,Ti)(Cr,Si,Ge)_2_ (green) and (Nb,Ti)(Cr,Si,Sn)_2_ (dark orange). In (**b**,**c**) the triangles are data for (Nb,Ti)(Cr,Si,Al,Sn)_2_ and (Nb,Ti,Mo,Hf)(Cr,Si,Al,Sn)_2_ (light orange), and (Nb,Ti)(Cr,Si,Al,Ge)_2_ (light green); the diamonds are data for (Nb,Mo)(Cr,Si,Al)_2_ (yellow), (Nb,Ti,Ta)(Cr,Si,Al)_2_, (Nb,Ti,Hf)(Cr,Si,Al)_2_, (Nb,Ti,Mo,Hf)(Cr,Si,Al)_2_ (red), (Nb,Ti,Hf)(Cr,Si,Ge)_2_ (light green), (Nb,Hf)(Cr,Si,Sn)_2_ and (Nb,Ti,Hf)(Cr,Si,Sn)_2_ (dark orange), (Nb,Ti,Mo,W,Hf)(Cr,Si,Sn)_2_ (brown). In (**a**–**c**) the black triangle represents the Nb–55Cr–15Si Laves phase.

The effects of substituting Nb and Cr by other elements are shown in the ∆χ versus VEC maps in Figure 2a,b. Figure 2a shows data for the unalloyed NbCr_2_ and alloyed Laves, where Cr is substituted by Si, Al, Ge or Sn, and Nb by Ti. The arrows in Figure 2a show the “direction of change” as Cr and Nb are substituted. There is a decrease in the value of VEC and a slight increase in the value of ∆χ (becomes less negative) when only Si substitutes Cr and a further shift towards lower VEC and less negative ∆χ values when Nb is substituted by Ti, and Cr by Si and Al, or Si and Ge, or Si and Sn. Figure 2b shows a similar map for “more heavily” alloyed Laves phases where more elements substitute Nb and Cr. The “direction of change” is indicated by the arrow and it should be noted that further alloying keeps the data to the left of Nb(Cr,Si)_2_ but within a band that is defined by dashed lines that are essentially parallel to that for the (Nb,Ti)(Cr,Si)_2_ Laves phase in Figure 2a. When the data for the C14–NbCr_2_ Laves phases is considered in the ∆χ versus R_<Nb>_/R_<Cr>_ map in Figure 2c, the data exhibits a remarkable linear fit with R^2^ = 0.9626 and shows that ∆χ becomes less negative as alloying shifts its values from the bottom of the line (occupied by the unalloyed NbCr_2_) towards the data for (Nb,Ti)(Cr,Si,Ge)_2_ and (Nb,Ti)(Cr,Si,Sn)_2_ at the top.

**Figure 3:**
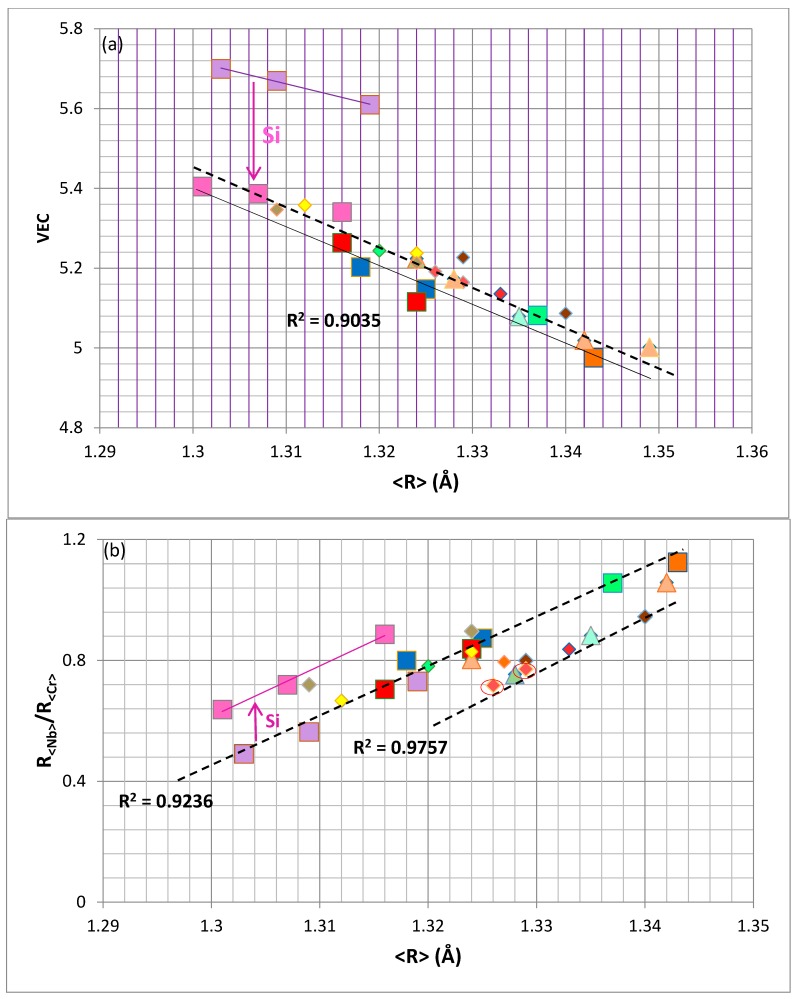
Laves phase maps (**a**) VEC versus <R> = R_<Nb>_ + R_<Cr>_ and (**b**) R_<Nb>_/R_<Cr>_ versus <R> = R_<Nb>_ + R_<Cr>_. Lavender, pink, blue, red, green and dark orange squares represent the same data as in Figure 2a. The triangles represent data for (Nb,Ti)(Cr,Si,Al,Sn)_2_ and (Nb,Ti,Mo,Hf)(Cr,Si,Al,Sn)_2_ (light orange), and (Nb,Ti)(Cr,Si,Al,Ge)_2_ (light green); diamonds represent data for (Nb,Mo)(Cr,Si,Al)_2_ (yellow), (Nb,Ti,Ta)(Cr,Si,Al)_2_, (Nb,Ti,Hf)(Cr,Si,Al)_2_, (Nb,Ti,Mo,Hf)(Cr,Si,Al)_2_ (red), (Nb,Ti,Hf)(Cr,Si,Ge)_2_ (light green), (Nb,Hf)(Cr,Si,Sn)_2_ and (Nb,Ti,Hf)(Cr,Si,Sn)_2_ (dark orange), (Nb,Ti,Mo,W,Hf)(Cr,Si,Sn)_2_ (brown).

The importance of atomic size on the alloying behaviour of NbCr_2_ also can be captured in maps of VEC versus <R> (=R_<Nb>_ + R_<Cr>_) and R_<Nb>_/R_<Cr>_ versus <R>, see Figure 3. Figure 3a shows the shift towards lower VEC values upon alloying NbCr_2_ with Si and further decrease in VEC with increasing <R> upon further alloying. All the data exhibits a reasonable linear fit, shown by the dashed line for which R^2^ = 0.9035, and remarkably the latter is essentially parallel to the line joining 35.5Nb–(52.5Cr–12Si) with (Nb,Ti)(Cr,Si)_2_ and Nb(Cr,Si,Sn)_2_ (thin continuous line in Figure 3a). Figure 3b shows the step change in R_<Nb>_/R_<Cr>_ of the unalloyed NbCr_2_ to higher values upon alloying with Si (see arrow) and the continuous increase of the ratio upon further substitution of Nb and Cr with other transition metals and simple metal and metalloid elements, the addition of which leads to an increase in <R>. This map can separate alloying behaviours, as shown by the two dashed lines, of which the lower one (R^2^ = 0.9757) corresponds to data for the Laves phases (Nb,Ti)(Cr,Si,Al,Ge)_2_, (Nb,Ti,Hf)(Cr,Si,Al)_2_, (Nb,Ti,Hf)(Cr,Si,Sn)_2_, (Nb,Ti,Mo,Hf)(Cr,Si,Al)_2_ and (Nb,Ti,Mo,Hf)(Cr,Si,Sn)_2_. The two encircled data points in the Figure 3b correspond to the average compositions of the Laves phase grains in the uncontaminated bulk of an alloy that was isothermally oxidised at 1073 and 1473 K.

**Figure 4:**
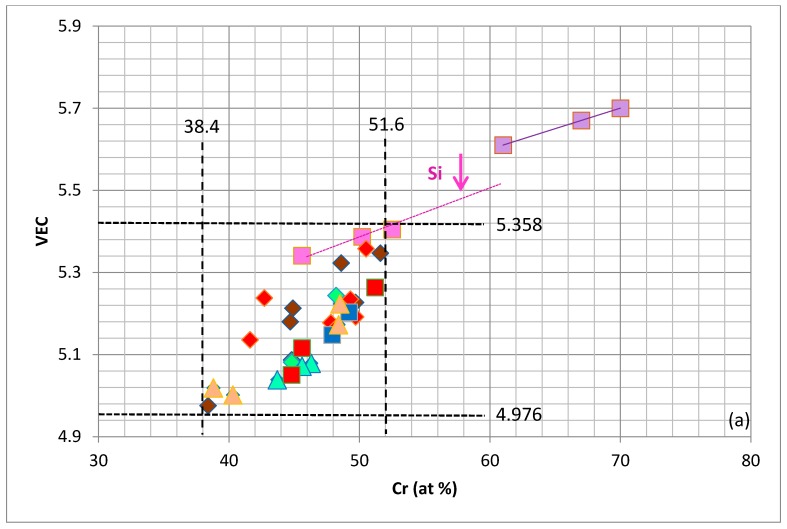

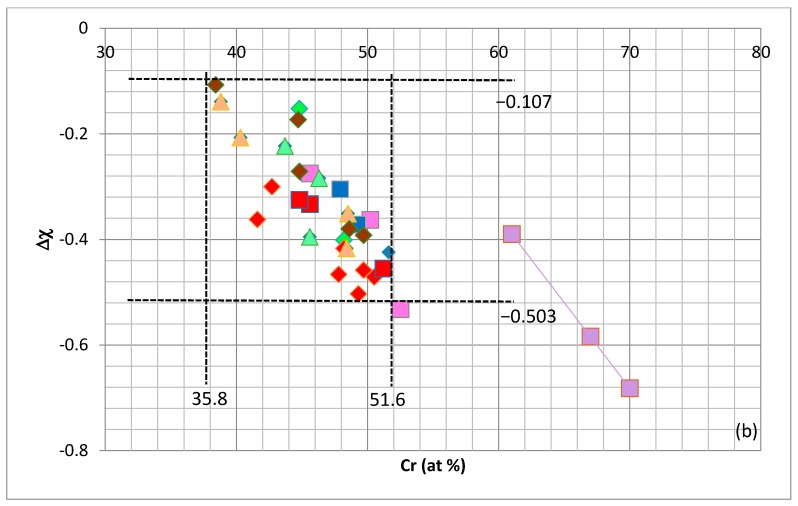
Laves phase maps of VEC (**a**) and ∆χ (**b**) versus Cr concentration in the Laves phase. Lavender, pink, blue, red, light green and dark orange squares represent the same data as in Figure 2a. Triangles represent (Nb,Ti)(Cr,Si,Al,Sn)_2_ and (Nb,Ti,Mo,Hf)(Cr,Si,Al,Sn)_2_ (light orange), (Nb,Ti)(Cr,Si,Al,Ge)_2_ (light green); diamonds represent (Nb,Mo)(Cr,Si,Al)_2_ (rose), (Nb,Ti,Ta)(Cr,Si,Al)_2_, (Nb,Ti,Hf)(Cr,Si,Al)_2_, (Nb,Ti,Mo,Hf)(Cr,Si,Al)_2_ (red), (Nb,Ti,Hf)(Cr,Si,Ge)_2_ (light green), (Nb,Hf)(Cr,Si,Sn)_2_ and (Nb,Ti,Hf)(Cr,Si,Sn)_2_ (dark orange), (Nb,Ti,Mo,W,Hf)(Cr,Si,Sn)_2_ (brown).

The changes in the values of the parameters VEC and ∆χ of NbCr_2_ upon “simple” and “advanced” alloying are shown in Figure 2a,b. The currently available data is summarised in Figure 4 and shows that the values of the parameters VEC and ∆χ of the alloyed Laves phases that are observed in Nb–silicide-based alloys are confined within well-defined ranges. The Laves phase parameters VEC and ∆χ, respectively, are restricted in the ranges, 4.976 < VEC < 5.358 and −0.503 < ∆χ < −0.107, while the Cr concentration is confined to the range 35.8 < Cr < 51.6 at %. The Laves parameter VEC is within the range of the VEC values of the bcc Nb solid solutions in Nb–silicide-based alloys [42].

### 2.2. A15–Nb*_3_*X

In Nb–silicide-based alloys, the A15–Nb_3_X phase(s) (X = Al, Ge, Si, Sn) can form in the cast microstructures and/or after heat treatment and/or during oxidation [3,4,43,48,52,53]. The Nb in A15–Nb_3_X may be substituted by other transition metals, such as Cr, Fe, Hf, Mo, Ti or W [3,4,43,48,52,53]. The elements Al, Cr, Fe, Ge, Hf and Sn are important for improving the oxidation of Nb–silicide-based alloys [2,56].

The solubility ranges of Al, Ge and Sn in unalloyed A15 phases, respectively, were 18.6 < Al < 25 at % for Nb_3_Al, 18 < Ge < 23 at % for Nb_3_Ge and 15.5 < Sn < 33.2 at % for Nb_3_Sn [15]. In the alloyed A15 phases observed in Nb–silicide-based alloys, the solubilities of these elements were 2.4 < Al < 8.9 at %, 1.1 < Ge < 3.8 at %, 5.2 < Sn < 16 at % and 1.4 < Si < 8.8 at %, and X (=Al + Ge + Si + Sn) in Nb_3_X was in the range 16.3 < X < 22.7 at %. Like the Laves phases, no strong relationships between the concentrations of alloying elements were found in A15–Nb_3_X intermetallic phases.

**Figure 5:**
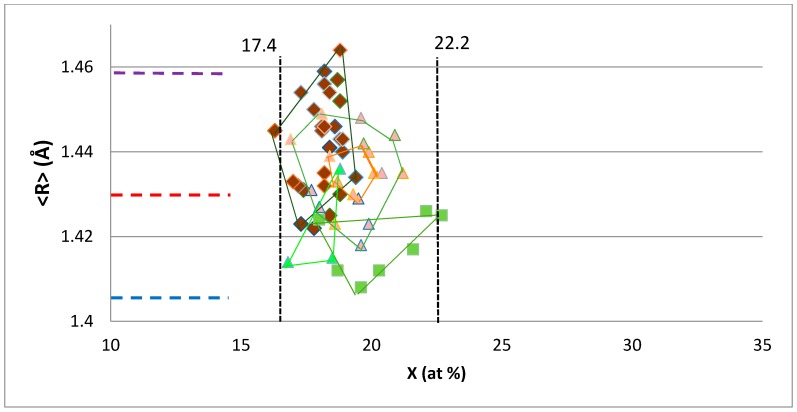
<R> versus concentration of X in A15–Nb_3_X where Nb is substituted by Cr, Fe, Hf, Mo, Ti, W, and X = Al, Ge, Si, Sn. Data for A15–Nb_3_X with X = Si, Sn is shown by brown diamonds, data for X = Si, Sn, Al with rose triangles, data for X = Ge, Si, Sn with green triangles and data for X = Al, Ge, Si or Sn with green squares. For vertical and dashed lines, see text.

Figure 5 shows that the A15–Nb_3_X phases that are formed in Nb–silicide-based alloys have X within the range defined by the averages of the minimum and total values, respectively, of unalloyed Nb_3_Al, Nb_3_Ge, Nb_3_Sn. Figure 5 also shows that the <R> of A15 phases in Nb–silicide-based alloys is within the area defined by the horizontal blue and purple dashed lines. The dashed lines from bottom (blue) to top (purple) correspond to the average <R> of unalloyed Nb_3_Al, Nb_3_Ge and Nb_3_Sn, and the <R> of Nb–25Al and Nb–15.5Sn. The <R> of Nb_3_X alloyed with Ge is confined between the blue and red dashed horizontal lines, i.e., between the average <R> of unalloyed Nb_3_Al, Nb_3_Ge, Nb_3_Sn, and the <R> of Nb–25Al, while the data for Nb_3_X alloyed with Al is on both sides of the red horizontal dashed line that is for the <R> of Nb–25Al.

The solubilities of the other elements in alloyed Nb_3_X where Nb is substituted by one or more of the elements Cr, Fe, Hf, Mo, Ti or W, and X is substituted by two or more of the elements Al, Ge, Si or Sn were as follows: 1.4 < Cr < 8.7 at %, Fe < 2.6 at %, Hf < 6.3 at %, 5.1 < Mo < 18.5 at %, 7.8 < Ti < 32.1 at % and 1.6 < W < 5.2 at %. Very Ti rich A15 phases can also form [52], in which case, 38.5 < Ti < 48.6 at %.

**Figure 6:**
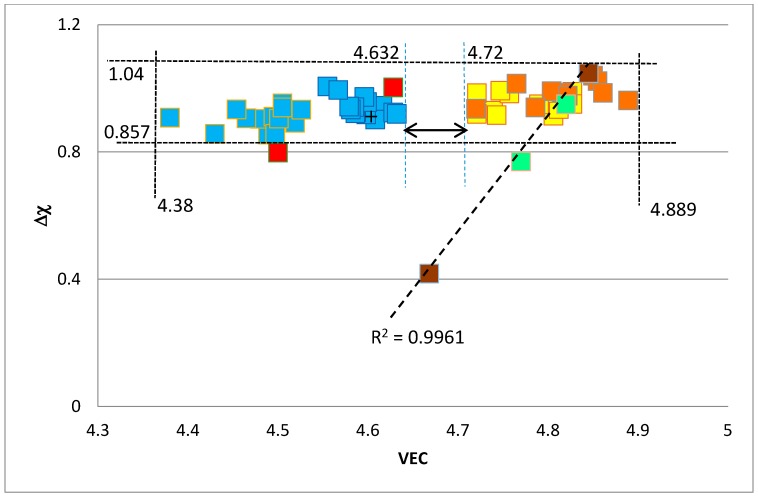
Map of ∆χ versus VEC of A15–Nb_3_X phases where Nb is substituted by Cr, Fe, Hf, Mo, Ti, W and X = Al, Ge, Si, Sn. Brown, red and green squares represent data for Nb_3_X with X = Sn, Al or Ge, respectively. Light brown squares represent data for Nb substituted by Cr, Hf, Mo, Ti, W and X = Al, Ge, Si, Sn, yellow squares represent data for Nb substituted by Cr, Hf, and X = Al, Si, Sn and blue squares represent data for Nb substituted by Cr, Fe, Hf, Ti and X = Al, Si, Sn.

The ranges of the values of the parameters ∆χ and VEC of alloyed A15–Nb_3_X are shown in the ∆χ versus VEC map in Figure 6 and are 0.857 < ∆χ < 1.04 and 4.38 < VEC < 4.889, respectively, with a gap in VEC values between 4.632 and 4.72. It should be noted that unalloyed Nb_3_Sn falls in the VEC gap, but in the latter, there are no alloyed A15–Nb_3_X phases. The values of both parameters fall within the ranges of ∆χ and VEC for bcc Nb solid solutions [42] with the lower and upper limits of VEC being essentially the same as those of the Nb_ss_. The values of the parameter VEC of alloyed A15–Nb_3_X phases are lower than those of C14–NbCr_2_ Laves phases.

The data for the unalloyed A15–Nb_3_Al, Nb_3_Ge and Nb_3_Sn are also shown in the ∆χ versus VEC map in Figure 6. It should be noted that A15–Nb_3_X phases, where Nb is substituted by Cr, Fe, Hf or Ti and X is Al, Si, Sn, are on the left-hand side of the VEC gap. Also in the gap is the data for unalloyed Nb_3_Al, while the data for the A15–Nb_3_X phases, where Nb is substituted by Cr and Hf, and X is Al, Si, Sn or Nb is substituted by Cr, Hf, Mo, Ti or W, and X is Al, Ge, Si, Sn, falls on the right-hand side of the VEC gap, where the data for unalloyed Nb_3_Sn and Nb_3_Ge is found. The data for Nb_3_Sn, Nb_3_Ge and Nb_3_(Si,Sn) exhibits a remarkably good linear fit with R^2^ = 0.9961.

The hardness of a material is related to both its elastic and plastic deformation as the indenter is applied perpendicularly to its surface and the sample is subjected to a combination of compression, shear and tension. Bulk properties are important for hardness because the indenter interacts with the surface and the bulk of the sample. The shear modulus provides a measure of the rigidity against the shape deformations as the force of the indenter is applied perpendicularly to the sample surface [57]. Jhi et al. [57] studied the hardness of transition metal carbides and nitrides and suggested that owing to their high Peierls stresses, their strengths may be influenced by the difficulty of nucleating and moving dislocations. The stresses required for the latter scale with the shear modulus and thus, electronic changes that affect the shear modulus, would have a strong effect on the hardness. They concluded that bonding may be important for the hardness of transition metal carbides and nitrides rather than the conventional microstructural features that determine the hardness of metals and alloys, and that the response of bonds to shear is the crucial factor in determining hardness. Furthermore, Jhi et al. [57] found a correlation between the calculated shear modulus c_44_, “which represents a shape change without volume change and provides directly information about electronic response to shear strain”, and VEC, and between the experimentally measured hardness values and VEC, for transition metal (Hf,Ti,Zr) carbonitrides and Nb carbides. A similar correlation between c_44_ and VEC was reported by Wang and Zhou for M_2_AlC, who also found that the bulk and shear moduli increased monotonously as VEC increased. They suggested that the hardness of M_2_AlC could be tuned by alloying to get “appropriate” VEC values [58]. A similar suggestion was made earlier by Konig for “tuning” the value of VEC via alloying and thus the properties of hard coatings [59].

To the author’s knowledge, data about the Peierls stress of A15 compounds is limited. The compound Nb_3_Al with the A15 structure is likely to have considerable Peierls stress [60]. Shyue’s conjecture about the Peierls stress of A15 compounds is supported by the recent work of Kamimura et al. who calculated the Peierls stress of the A15–V_3_Si intermetallic to be about 13,500 MPa, compared with 6500 MPa for TiC, 415 MPa for Nb, and less than 1.4 MPa for Al [61]. It would be interesting to find out if there is a relationship between the hardness of A15–Nb_3_X in Nb–silicide-based alloys and its parameter VEC. This will be addressed below.

Pauling defined electronegativity as “the power of an atom in a molecule to attract electrons to itself” [62]. Electronegativity provides primary information about bonding. The importance of bonding for the hardness of materials was also discussed by Li et al. who proposed an empirical model to predict the hardness of materials in terms of electronegativity and crystal structure [63]. Is there a relationship between the hardness of A15–Nb_3_X in Nb–silicide-based alloys and their electronegativities?

**Figure 7:**
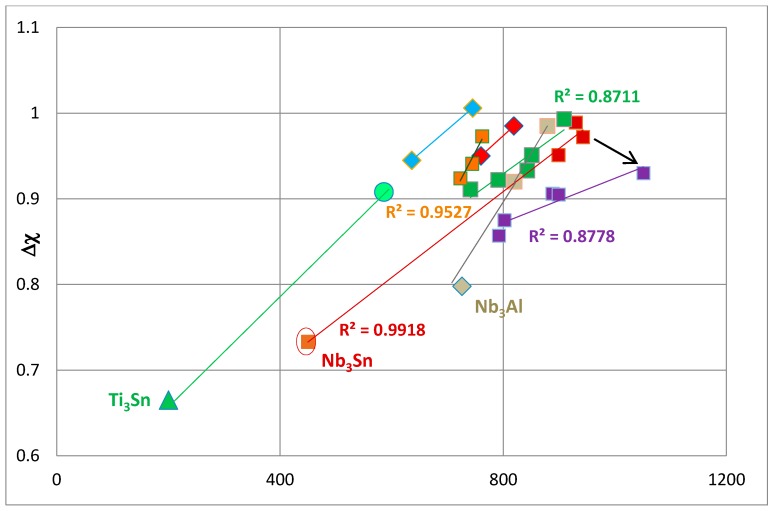
∆χ versus Vickers hardness (HV) of A15–Nb_3_X and Ti_3_Sn. Dark red squares represent data for Nb_3_Sn and Nb_3_(Sn,Si); the encircled data point represents unalloyed Nb_3_Sn, green triangles represent unalloyed Ti_3_Sn, tan diamonds represent unalloyed Nb_3_Al, green squares represent (Nb,Ti,Hf)_3_(Si,Sn), (Nb,Cr,Hf)_3_(Si,Sn) and (Nb,Ti,Cr,Hf)_3_(Si,Sn), blue diamonds represent (Nb,Ti)_3_(Si,Sn), red diamonds represent (Nb,Hf)_3_(Si,Sn), orange squares represent (Nb,Ti,Fe)_3_(Si,Sn), purple squares represent (Nb,Ti,Hf)_3_(Si,Sn,Al) and (Nb,Ti,Cr,Hf)_3_(Si,Sn,Al), tan squares represent (Nb,Hf)_3_(Si,Sn,Al) and green circles represent Ti rich (Nb,Ti,Fe)_3_(Si,Sn).

**Figure 8:**
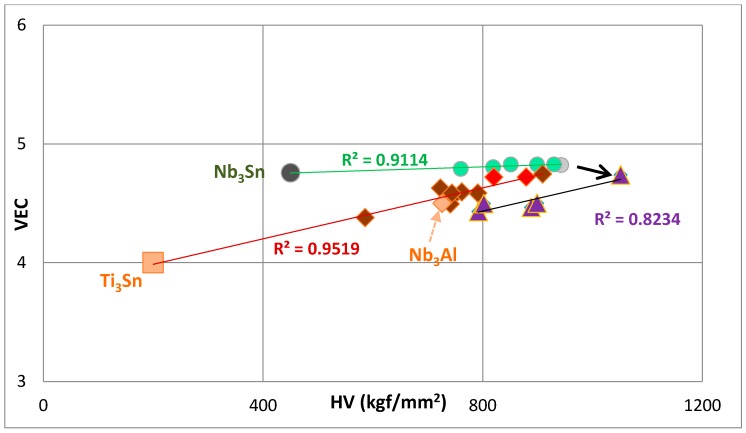
VEC versus hardness (HV) of A15–Nb_3_X and Ti_3_Sn (light orange square). Green circles represent Nb_3_Sn (dark green), Nb_3_(Sn,Si) (very light green), (Nb,Hf)_3_(Sn,Si) and (Nb,Hf,Cr)_3_(Sn,Si). Dark red diamonds represent (Nb,Ti)_3_(Sn,Si), (Nb,Hf)_3_(Sn,Si), (Nb,Cr,Hf)_3_(Sn,Si), (Nb, Ti,Cr,Hf)_3_(Sn,Si), (Nb,Ti,Fe)_3_(Sn,Si), (Nb,Hf)_3_(Sn,Si,Al) (red diamonds); the light orange diamond represents Nb_3_Al, and purple triangles represent Nb_3_(Sn,Si,Al), (Nb,Ti,Hf)_3_(Sn,Si,Al) and (Nb,Ti,Hf,Cr)_3_(Sn,Si,Al).

The available Vickers hardness (HV) data [4,48,52] for unalloyed and alloyed A15–Nb_3_X phases is shown in Figure 7 and Figure 8, where the hardness is plotted against the parameters ∆χ and VEC, respectively. The calculated and measured hardness values of Nb_3_X (X = Al, Sn) are shown in Table 1. There is good agreement between measured values of Nb_3_Sn and Nb_3_Al and one of the calculated values (indicated in bold), based on Young’s modulus E and Poisson’s ratio ν, or Bulk modulus B and shear modulus G. Interestingly, the calculated hardness values using only the macroscopic shear modulus G are significantly higher than the measured hardness. The data in Figure 7 and Figure 8 is separated into groups. The goodness of linear fit is indicated by the R^2^ values. Data points and R^2^ values are given using the same colour for a particular set of data. The hardness values of Nb_3_Sn, Ti_3_Sn and Nb_3_Al, respectively, are taken from [4,52,64]. The structure of Ti_3_Sn is DO_19_ with prototype Ni_3_Sn [15]. Figure 7 and Figure 8 show that the hardness values of alloyed A15–Nb_3_X phases are correlated with their parameters, ∆χ and VEC, and that an increase in the values of the latter is accompanied by an increase in hardness.

Figure 7 shows (a) that the Ti_3_Sn has lower ∆χ and hardness than Nb_3_Sn, which has a lower ∆χ and hardness than Nb_3_Al and (b) that upon alloying, the ∆χ values of A15–A_3_X phases (A = Nb, Ti, X = Al, Sn) increase and these increases are accompanied by increases in hardness. The alloyed A15–Nb_3_X phases have hardness values above 600 HV and lower than 1100 HV. Substitution of Sn by Si in Nb_3_Sn results in significant increases in both ∆χ and the hardness of Nb_3_(Si,Sn) (red squares). When Nb is substituted by Hf in (Nb,Hf)_3_(Si,Sn) (red diamonds), the hardness decreases, but the slope remains the same, and substitution of Nb by Ti in (Nb,Ti)_3_(Si,Sn) (blue diamonds) results in a further decrease in hardness but with no change in slope. When Nb is substituted by both Hf and Ti, or Cr and Hf or Gr, Hf and Ti, the data (green squares) moves to the right, closer, but not exceeding, the data for Nb_3_(Si,Sn) (red squares) and with essentially the same slope, from which it can be deduced that the substitution of Nb by Cr has the same effect on ∆χ and hardness as Hf or Ti, meaning the (Nb,Cr)_3_(Si,Sn) moves to the left of the Nb_3_(Si,Sn) data with no change in slope. In other words, alloying with Hf or Ti or Hf and Ti, or Cr and Hf or Cr, Hf and Ti moves the data for alloyed A15–Nb_3_(Si,Sn) parallel to the line for Nb_3_(Si,Sn) with small changes in ∆χ values and small or significant (in the case of Ti or Hf) changes in hardness, particularly for (Nb,Ti)_3_(Si,Sn).

The substitution of Nb by Fe and Ti in (Nb,Fe,Ti)_3_(Si,Sn) also results in a shift to the left of the data for Nb_3_(Si,Sn) but with a higher slope (orange squares) which would suggest that the simultaneous presence of Fe and Ti affects ∆χ (and thus the hardness) differently than that of Hf and Ti. Note that the data point for the Ti-rich (Nb,Fe,Ti)_3_(Si,Sn) falls on a line with the same slope as the data for (Nb,Ti)_3_(Si,Sn) and that the data for unalloyed Ti_3_Sn, (Nb,Fe,Ti)_3_(Si,Sn) and (Nb,Ti)_3_(Si,Sn) has the same slope but the values of ∆χ and hardness are lower as the Ti concentration in the A15 phase increases. This confirms that the increase in the concentration of Ti in the A15 phases is accompanied by reductions of both ∆χ and hardness.

The unalloyed Nb_3_Al is on the right-hand side of the Nb_3_(Si,Sn) line. The addition of Al in Nb_3_(Si,Sn,Al) results in a reduction of ∆χ and an increase in hardness (see arrow in Figure 7). The Nb_3_(Si,Sn,Al) has the highest hardness of the A15–Nb_3_X phases. When Al is added to (Nb,Hf)_3_(Si,Sn,Al) (tan squares), the resultant changes in ∆χ and hardness change the slope of the line, the extension of which passes from the data point for unalloyed Nb_3_Al. Indeed, when the data for Nb_3_Al is joined with the data for (Nb,Hf)_3_(Si,Sn,Al) (tan squares), R^2^ = 0.9982. The data also shows that when the Al in Nb_3_Al is substituted by Si and Sn, and the Nb by Hf, both the ∆χ and hardness increase. Furthermore, the line for (Nb,Hf)_3_(Si,Sn,Al) (tan squares) is to the left of the line for Nb_3_(Si,Sn) (red squares), which confirms the effect of Hf on hardness, discussed above, but it is to the right of the line for (Nb,Hf)_3_(Si,Sn) (red diamonds) owing to the effect that Al has on the hardness (see above). Interestingly, when Ti and Hf, or Ti, Hf and Cr are simultaneously present with Al in A15-phases (purple squares), the ∆χ is reduced, the hardness hardly changes and thus, the slope is reduced. The changes shown by the purple data in Figure 7 are attributed to the strong role played by Al in A15–Nb_3_X phases and the synergy of Al with the aforementioned transition metals (compared with data presented by the green and purple squares).

It should be noted that the limited data for Fe-containing A15–Nb_3_X phases (orange squares) has the same slope as the line for unalloyed Nb_3_Al (tan diamond) and (Nb,Hf)_3_(Si,Sn) (tan squares), which may be an indication that the substitution of Nb by Fe, and Ti has the same effect on the slope d(∆χ)/d(HV) as the substitution of Nb by Hf and the addition of Al. Unfortunately, there is no data for (Nb,Ti,Fe)_3_(Si,Sn,Al) A15 phases to test this hypothesis.

Figure 8 shows how the hardness of A15–Nb_3_X phases changes with their parameter, VEC. The latter cannot separate the contributions of different elements, for example Hf, as effectively as the parameter ∆χ. The Ti_3_Sn (light orange square) has a lower VEC than unalloyed Nb_3_Al (light orange diamond), which has a lower VEC than unalloyed Nb_3_Sn (dark green circle), and the Ti_3_Sn and unalloyed Nb_3_Al are on the same line (red diamonds) as data for A15–Nb_3_X phases where X = Si, Sn and Nb are substituted by Hf, or Ti, or Hf and Cr, or Ti and Cr, or Ti and Fe; they are also on the same line as the data for (Nb,Hf)_3_(Si,Sn,Al) (red diamonds). Furthermore, the extension of the line fitted to the data presented by the diamonds crosses the data point for Nb_3_(Si,Sn) (very light green circle). When Ti or Ti and Cr are added to (Nb,Hf)_3_(Si,Sn,Al), there is a reduction in VEC and the slope d(VEC)/d(HV) is not changed (purple triangles).

In Figure 8, the data represented by the circles is for A15 phases without Ti. This element is present in some of the A15 phases, the data points of which are on the line fitted to the red diamonds; this confirms that Ti reduces the VEC of alloyed A15–Nb_3_X phases. The addition of Al in A15 phases is again seen to increase the hardness (see arrow in Figure 8) and to decrease VEC. Thus, the VEC versus hardness map also confirms that the substitution of Sn by Si increases the hardness significantly but not the value of the parameter VEC, while the addition of Al decreases VEC significantly and increases the hardness but not as much as Si.

**Figure 9:**
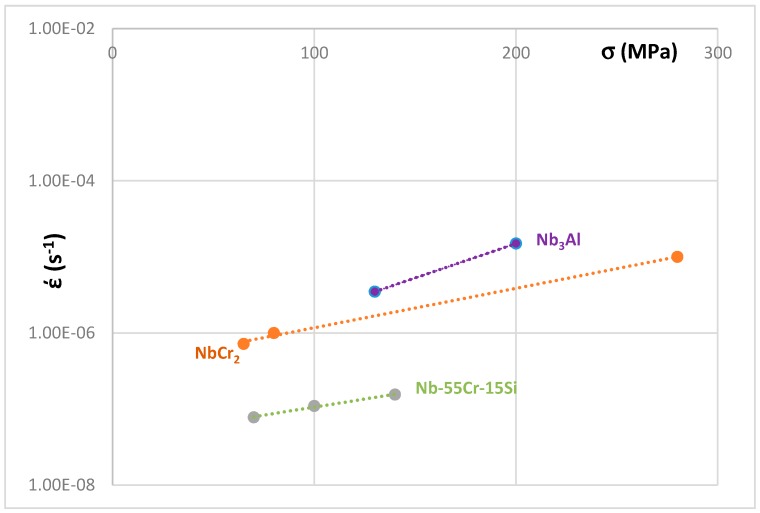
Norton plot of creep rate (s^−1^) versus stress (MPa) at 1473 K of unalloyed and Si alloyed Laves phases [18,52] and unalloyed A15–Nb_3_Al [18]. The former are shown in the ∆χ versus VEC maps in Figure 2a,b and the latter in the map in Figure 6 (see text).

The creep at 1473 K of unalloyed A15–Nb_3_Al and unalloyed and Si alloyed NbCr_2_ Laves phases is compared in Figure 9. The creep data is from [23,55]. The Laves phases are shown by the lavender squares and black triangles in Figure 2 and A15–Nb_3_Al is shown by the cross in the red square in Figure 6. The improvement of the creep of the alloyed Laves phase in Figure 9 is related to a decrease of VEC and ∆χ. The experimental data for the creep of alloyed C14 NbCr_2_ Laves phases is more limited compared with the data for the creep of Nb_5_Si_3_ [41]. For the latter silicide, the creep rate έ increased with alloying (excluding Boron addition) and the increase of έ was related to a decrease in VEC and increase in ∆χ. However, for the Nb_5_Si_3_ alloyed with Boron, the increase in έ was related to decreases in both the parameters VEC and ∆χ.

## 3. Conclusions

In this paper, the alloying behaviours of the hexagonal C14 Laves and cubic A15 intermetallic phases in Nb–silicide-based alloys were studied using the parameters VEC, ∆χ and atomic size. Experimental data for Laves phases where Nb is substituted by Hf, Mo, Ta, Ti or W and Cr by Al, Ge, Si or Sn, and A15 phases where Nb is substituted by Cr, Fe, Hf, Mo, Ta, Ti or W and Sn by Al, Ge or Si, were used for the calculations. The conclusions of the research are as follows:

For the Laves phase, the parameters VEC and ∆χ were in the ranges 4.976 < VEC < 5.358 and −0.503 < ∆χ < −0.107, the Cr concentration was in the range 35.8 < Cr < 51.6 at %, compared with 61 < Cr < 70 at % in the unalloyed NbCr_2_, and the Cr + Al + Ge + Si + Sn sum was in the range 50.9 to 64.5 at %.

The maps of ∆χ versus Cr, ∆χ versus VEC and VEC versus <R> = R_<Nb>_ + R_<Cr>_ could separate the alloying behaviours of different elements in the Laves phase. Compared with the unalloyed NbCr_2_, the parameter VEC was decreased and ∆χ increased (became less negative) when only Si substituted Cr, and the changes of both parameters increased when Nb was substituted by Ti, and Cr by Si and Al, or Si and Ge, or Si and Sn.

For the A15 phase, the parameters VEC and ∆χ were in the ranges 4.38 < VEC < 4.89 and 0.857 < ∆χ < 1.04 with a gap in the VEC values between 4.63 and 4.72; the concentration of the elements Al + Ge + Si + Sn was in the range 16.3 to 22.7 at % and the range of each individual element was 2.4 < Al < 8.9 at %, 1.1 < Ge < 3.8 at %, 1.4 < Si < 8.8 at % and 5.2 < Sn < 16 at %. The VEC versus ∆χ map could separate the alloying behaviours of the elements in the A15 intermetallic phases.

The hardness of A15 Nb_3_X was correlated with the parameters ∆χ and VEC. An increase in the latter was accompanied by an increase in hardness.

The substitution of Sn by Si in Nb_3_Sn resulted in significant increases of the ∆χ and hardness values of Nb_3_(Si,Sn), but the parameter VEC hardly changed, while the addition of Al decreased VEC significantly and increased the hardness but not as much as for Si. The substitution of Nb by Cr had the same effect on ∆χ and hardness as Hf or Ti, and an increase in the concentration of Ti in A15 decreased both the ∆χ and hardness. The addition of Al in Nb_3_(Si,Sn,Al) decreased ∆χ and increased the hardness, but when Ti and Hf, or Ti, Hf and Cr were simultaneously present with Al, the parameter ∆χ was decreased and the hardness hardly changed.

The improvement in the creep of the Si alloyed Laves phase was associated with decreases in VEC and ∆χ.

## Figures and Tables

**Table 1:** Vickers hardness (HV) (kgf/mm^2^) of unalloyed Nb_3_X (X = Al, Sn). The HV*, HV^+^ and HV^C^ were calculated in GPa using HV* = (1 − 2ν)E/[6(1 + ν)], HV^+^ = 2[(G/B)^2^ G]^0.585^ − 3 and HV^C^ = 0.151G [65], and the data for B, E, G and ν from [30,31,32] and were converted to Vickers hardness **.

Phase	HV*	HV^+^	HV^C^	HV_measured_
Nb_3_Sn	760	469	1002	450
Nb_3_Al	708	427	949	726

** To convert HV to GPa, multiply by 0.009807.
